# Supplementary material for: A deep dive into the use of local positioning system in professional handball: Automatic detection of players’ orientation, position and game phases to analyse specific physical demands
Source: PLoS One. 2023 Aug 16;18(8):e0289752. doi: 10.1371/journal.pone.0289752 (PMC10431627; doi:10.1371/journal.pone.0289752)
Supplement: S1 Table — (DOCX) [file pone.0289752.s001.docx]

**S1 Table. Descriptive statistics per playing position.**

| Phase |  | Time  (min) | Distance  (m) | Accel’Rate  (u.a.) | Normalised  distance (m/10min) | Normalised  Accel’Rate (u.a./10min) |
| --- | --- | --- | --- | --- | --- | --- |
| Versatile back players (N = 160) | N | 160 | 160 | 153 | 160 | 153 |
|  | Mean | 27.64 | 2313.97 | 238.07 | 842.83 | 86.18 |
|  | SD | 8.44 | 681.12 | 79.67 | 76.62 | 11.48 |
| Offesnive back players (N = 33) | N | 33 | 33 | 33 | 33 | 33 |
|  | Mean | 15.06 | 1634.52 | 174.89 | 1105.02 | 118.09 |
|  | SD | 6.39 | 631.94 | 67.68 | 84.86 | 11.46 |
| Goalkeepers (N=40) | N | 40 | 40 | 39 | 40 | 39 |
|  | Mean | 49.43 | 2008.03 | 133.58 | 405.58 | 26.87 |
|  | SD | 16.70 | 702.46 | 44.43 | 28.38 | 3.32 |
| Versatile line players (N=73) | N | 73 | 73 | 73 | 73 | 73 |
|  | Mean | 29.97 | 2380.24 | 244.56 | 793.58 | 82.41 |
|  | SD | 10.09 | 829.82 | 80.56 | 60.33 | 9.02 |
| Defensive Position 3 players (N=37) | N | 37 | 37 | 37 | 37 | 37 |
|  | Mean | 30.11 | 2062.45 | 214.24 | 688.13 | 71.529 |
|  | SD | 8.75 | 590.72 | 63.97 | 47.81 | 7.926 |
| Wing players (N = 120) | N | 120 | 120 | 97 | 120 | 97 |
|  | Mean | 32.30 | 2925.81 | 297.01 | 911.38 | 92.00 |
|  | SD | 11.58 | 998.79 | 102.21 | 63.27 | 12.49 |

|  |  | Standing | | Walking | | Jogging | | Running | | Sprinting | |
| --- | --- | --- | --- | --- | --- | --- | --- | --- | --- | --- | --- |
|  |  | **% of time** | **Dist (m)** | **% of time** | **Dist (m)** | **% of time** | **Dist (m)** | **% of time** | **Dist (m)** | **% of time** | **Dist (m)** |
| Versatile back players (N = 160) | Mean | 6.12 | 12 | 72.14 | 1070 | 15.50 | 734 | 5.16 | 389 | 1.07 | 109 |
|  | SD | 1.94 | 6 | 2.87 | 337 | 2.35 | 209 | 1.25 | 132 | 0.49 | 58 |
| Offesnive back players (N = 33) | Mean | 3.22 | 4 | 63.05 | 604 | 23.67 | 597 | 8.15 | 327 | 1.90 | 103 |
|  | SD | 0.96 | 2 | 4.58 | 279 | 3.98 | 212 | 1.57 | 119 | 0.78 | 55 |
| Goalkeepers (N=40) | Mean | 19.17 | 61 | 77.54 | 1664 | 2.83 | 222 | 0.39 | 51 | 0.06 | 11 |
|  | SD | 3.21 | 21 | 3.39 | 609 | 0.79 | 76 | 0.16 | 25 | 0.05 | 10 |
| Versatile line players (N=73) | Mean | 6.58 | 15 | 74.12 | 1130 | 13.06 | 683 | 5.06 | 423 | 1.17 | 129 |
|  | SD | 1.80 | 6 | 2.18 | 384 | 1.60 | 244 | 1.15 | 177 | 0.43 | 63 |
| Defensive Position 3 players (N=37) | Mean | 8.06 | 18 | 76.81 | 1159 | 11.69 | 590 | 3.07 | 254 | 0.38 | 42 |
|  | SD | 1.69 | 7 | 2.24 | 333 | 1.76 | 173 | 0.79 | 98 | 0.18 | 25 |
| Wing players (N = 120) | Mean | 9.21 | 21 | 66.98 | 1063 | 12.94 | 738 | 7.41 | 664 | 3.47 | 438 |
|  | SD | 3.01 | 12 | 3.68 | 384 | 1.76 | 262 | 1.25 | 243 | 0.90 | 187 |

|  |  | Forward displacement | | Backward displacement | | Left displacement | | Right displacement | |
| --- | --- | --- | --- | --- | --- | --- | --- | --- | --- |
|  |  | **% of time** | **Dist (m)** | **% of time** | **Dist (m)** | **% of time** | **Dist (m)** | **% of time** | **Dist (m)** |
| Versatile back players (N =160) | Mean | 40.91 | 1091 | 15.37 | 300 | 18.58 | 344 | 25.14 | 584 |
|  | SD | 5.96 | 384 | 2.48 | 127 | 4.05 | 149 | 4.68 | 229 |
| Offesnive back players (N = 33) | Mean | 44.36 | 812 | 11.63 | 150 | 26.44 | 243 | 17.57 | 429 |
|  | SD | 6.95 | 363 | 2.78 | 84 | 7.39 | 107 | 3.85 | 215 |
| Goalkeepers (N=39) | Mean | 37.79 | 913 | 17.29 | 382 | 23.99 | 334 | 20.94 | 405 |
|  | SD | 3.61 | 337 | 1.74 | 148 | 2.77 | 133 | 2.36 | 162 |
| Versatile line players (N=73) | Mean | 39.36 | 1087 | 14.76 | 329 | 24.81 | 411 | 21.07 | 553 |
|  | SD | 5.89 | 479 | 3.59 | 176 | 2.87 | 166 | 3.79 | 202 |
| Defensive Position 3 players (N=37) | Mean | 39.99 | 989 | 12.70 | 206 | 25.85 | 343 | 21.46 | 525 |
|  | SD | 4.09 | 327 | 1.09 | 68 | 3.69 | 118 | 2.09 | 194 |
| Wing players (N = 97) | Mean | 39.31 | 1425 | 14.94 | 330 | 24.30 | 496 | 21.44 | 718 |
|  | SD | 6.92 | 587 | 3.40 | 186 | 4.97 | 239 | 3.65 | 343 |

N, number of players; SD, standard deviation; dist, distance covered.
