# Supplementary material for: A deep dive into the use of local positioning system in professional handball: Automatic detection of players’ orientation, position and game phases to analyse specific physical demands
Source: PLoS One. 2023 Aug 16;18(8):e0289752. doi: 10.1371/journal.pone.0289752 (PMC10431627; doi:10.1371/journal.pone.0289752)
Supplement: S2 Table — (DOCX) [file pone.0289752.s002.docx]

**S2 Table. Dunn's post-hoc test for the variable Distance normalised for positions factor.**

|  | | | | | | | | | | | | | |
| --- | --- | --- | --- | --- | --- | --- | --- | --- | --- | --- | --- | --- | --- |
| **Comparison** | | **z** | | **W _i_** | | **W _j_** | | **p** | | **p _bonf_** | | **p _holm_** | |
| Backs_Both - Backs_Off |  | -8.120 |  | 234.866 |  | 442.576 |  | < .001 | *** | < .001 | *** | < .001 | *** |
| Backs_Both - Goalkeeper |  | 9.063 |  | 234.866 |  | 20.500 |  | < .001 | *** | < .001 | *** | < .001 | *** |
| Backs_Both - Pivot_Both |  | 3.368 |  | 234.866 |  | 171.219 |  | < .001 | *** | 0.006 | ** | < .001 | *** |
| Backs_Both - Pos3_Def |  | 6.670 |  | 234.866 |  | 72.054 |  | < .001 | *** | < .001 | *** | < .001 | *** |
| Backs_Both - Wings |  | -5.706 |  | 234.866 |  | 327.063 |  | < .001 | *** | < .001 | *** | < .001 | *** |
| Backs_Off - Goalkeeper |  | 13.414 |  | 442.576 |  | 20.500 |  | < .001 | *** | < .001 | *** | < .001 | *** |
| Backs_Off - Pivot_Both |  | 9.668 |  | 442.576 |  | 171.219 |  | < .001 | *** | < .001 | *** | < .001 | *** |
| Backs_Off - Pos3_Def |  | 11.565 |  | 442.576 |  | 72.054 |  | < .001 | *** | < .001 | *** | < .001 | *** |
| Backs_Off - Wings |  | 4.392 |  | 442.576 |  | 327.063 |  | < .001 | *** | < .001 | *** | < .001 | *** |
| Goalkeeper - Pivot_Both |  | -5.726 |  | 20.500 |  | 171.219 |  | < .001 | *** | < .001 | *** | < .001 | *** |
| Goalkeeper - Pos3_Def |  | -1.689 |  | 20.500 |  | 72.054 |  | 0.046 | * | 0.684 |  | 0.046 | * |
| Goalkeeper - Wings |  | -12.549 |  | 20.500 |  | 327.063 |  | < .001 | *** | < .001 | *** | < .001 | *** |
| Pivot_Both - Pos3_Def |  | 3.673 |  | 171.219 |  | 72.054 |  | < .001 | *** | 0.002 | ** | < .001 | *** |
| Pivot_Both - Wings |  | -7.847 |  | 171.219 |  | 327.063 |  | < .001 | *** | < .001 | *** | < .001 | *** |
| Pos3_Def - Wings |  | -10.135 |  | 72.054 |  | 327.063 |  | < .001 | *** | < .001 | *** | < .001 | *** |
|  | | | | | | | | | | | | | |
| * p < .05, ** p < .01, *** p < .001 | | | | | | | | | | | | | |
