# Supplementary material for: A deep dive into the use of local positioning system in professional handball: Automatic detection of players’ orientation, position and game phases to analyse specific physical demands
Source: PLoS One. 2023 Aug 16;18(8):e0289752. doi: 10.1371/journal.pone.0289752 (PMC10431627; doi:10.1371/journal.pone.0289752)
Supplement: S3 Table — (DOCX) [file pone.0289752.s003.docx]

**S3 Table. Dunn's post-hoc test for the variable Accel’Rate normalised for positions factor.**

|  | | | | | | | | | | | | | |
| --- | --- | --- | --- | --- | --- | --- | --- | --- | --- | --- | --- | --- | --- |
| **Comparison** | | **z** | | **W _i_** | | **W _j_** | | **p** | | **p _bonf_** | | **p _holm_** | |
| Backs_Both - Backs_Off |  | -7.404 |  | 228.850 |  | 406.273 |  | < .001 | *** | < .001 | *** | < .001 | *** |
| Backs_Both - Goalkeeper |  | 9.325 |  | 228.850 |  | 20.000 |  | < .001 | *** | < .001 | *** | < .001 | *** |
| Backs_Both - Pivot_Both |  | 2.042 |  | 228.850 |  | 192.575 |  | 0.021 | * | 0.308 |  | 0.021 | * |
| Backs_Both - Pos3_Def |  | 5.542 |  | 228.850 |  | 102.095 |  | < .001 | *** | < .001 | *** | < .001 | *** |
| Backs_Both - Wings |  | -2.731 |  | 228.850 |  | 273.108 |  | 0.003 | ** | 0.047 | * | 0.006 | ** |
| Backs_Off - Goalkeeper |  | 13.080 |  | 406.273 |  | 20.000 |  | < .001 | *** | < .001 | *** | < .001 | *** |
| Backs_Off - Pivot_Both |  | 8.160 |  | 406.273 |  | 192.575 |  | < .001 | *** | < .001 | *** | < .001 | *** |
| Backs_Off - Pos3_Def |  | 10.175 |  | 406.273 |  | 102.095 |  | < .001 | *** | < .001 | *** | < .001 | *** |
| Backs_Off - Wings |  | 5.293 |  | 406.273 |  | 273.108 |  | < .001 | *** | < .001 | *** | < .001 | *** |
| Goalkeeper - Pivot_Both |  | -6.969 |  | 20.000 |  | 192.575 |  | < .001 | *** | < .001 | *** | < .001 | *** |
| Goalkeeper - Pos3_Def |  | -2.865 |  | 20.000 |  | 102.095 |  | 0.002 | ** | 0.031 | * | 0.006 | ** |
| Goalkeeper - Wings |  | -10.692 |  | 20.000 |  | 273.108 |  | < .001 | *** | < .001 | *** | < .001 | *** |
| Pivot_Both - Pos3_Def |  | 3.591 |  | 192.575 |  | 102.095 |  | < .001 | *** | 0.002 | ** | < .001 | *** |
| Pivot_Both - Wings |  | -4.163 |  | 192.575 |  | 273.108 |  | < .001 | *** | < .001 | *** | < .001 | *** |
| Pos3_Def - Wings |  | -7.089 |  | 102.095 |  | 273.108 |  | < .001 | *** | < .001 | *** | < .001 | *** |
|  | | | | | | | | | | | | | |
| * p < .05, ** p < .01, *** p < .001 | | | | | | | | | | | | | |
