# Supplementary material for: A deep dive into the use of local positioning system in professional handball: Automatic detection of players’ orientation, position and game phases to analyse specific physical demands
Source: PLoS One. 2023 Aug 16;18(8):e0289752. doi: 10.1371/journal.pone.0289752 (PMC10431627; doi:10.1371/journal.pone.0289752)
Supplement: S4 Table — (DOCX) [file pone.0289752.s004.docx]

**S4 Table. Descriptive statistics per game phases.**

| Phase |  | Time (min) | Distance (m) | Accel’Rate (u.a.) | Normalised distance (m/10min) | Normalised  Accel’Rate (u.a./10min) |
| --- | --- | --- | --- | --- | --- | --- |
| Defenive (N=374) | N | 404 | 404 | 374 | 404 | 374 |
|  | Mean | 16.67 | 930.58 | 94.38 | 558.82 | 56.47 |
|  | SD | 6.89 | 394.97 | 42.05 | 53.88 | 9.00 |
| Offensive (N=359) | N | 389 | 389 | 359 | 389 | 359 |
|  | Mean | 6.92 | 590.57 | 62.33 | 870.34 | 92.45 |
|  | SD | 3.86 | 317.89 | 34.99 | 145.68 | 20.67 |
| Defensive transition (N=393) | N | 423 | 423 | 393 | 423 | 393 |
|  | Mean | 2.38 | 376.58 | 36.53 | 1604.51 | 154.34 |
|  | SD | 1.12 | 182.46 | 20.89 | 319.97 | 45.22 |
| Offensive  Transition (N=392) | N | 422 | 422 | 392 | 422 | 392 |
|  | Mean | 3.51 | 590.55 | 61.29 | 1671.26 | 174.53 |
|  | SD | 1.45 | 264.93 | 29.01 | 242.02 | 35.22 |

|  |  | Standing | | Walking | | Jogging | | Running | | Sprinting | |
| --- | --- | --- | --- | --- | --- | --- | --- | --- | --- | --- | --- |
|  |  | **% of time** | **Dist (m)** | **% of time** | **Dist (m)** | **% of time** | **Dist (m)** | **% of time** | **Dist (m)** | **% of time** | **Dist (m)** |
| Defensive (N=374) | N | 404 | 404 | 404 | 404 | 404 | 404 | 403 | 403 | 384 | 384 |
|  | Mean | 8.46 | 11 | 83.08 | 645 | 6.68 | 183 | 1.36 | 64 | 0.45 | 30 |
|  | SD | 2.52 | 6 | 2.93 | 264 | 1.51 | 89 | 0.74 | 47 | 0.47 | 37 |
| Offensive (N=359) | N | 389 | 389 | 389 | 389 | 389 | 389 | 389 | 389 | 384 | 384 |
|  | Mean | 8.08 | 4 | 69.26 | 274 | 16.38 | 186 | 5.02 | 94 | 1.26 | 34 |
|  | SD | 6.40 | 5 | 6.83 | 154 | 5.87 | 106 | 1.76 | 55 | 0.86 | 30 |
| Defensive transition (N=393) | N | 410 | 410 | 423 | 423 | 423 | 423 | 423 | 423 | 416 | 416 |
|  | Mean | 1.93 | 0 | 40.66 | 69 | 34.90 | 146 | 17.34 | 112 | 5.30 | 50 |
|  | SD | 1.87 | 1 | 13.07 | 41 | 7.69 | 63 | 7.92 | 73 | 4.30 | 55 |
| Offensive  Transition (N=392) | N | 419 | 419 | 422 | 422 | 422 | 422 | 422 | 422 | 422 | 422 |
|  | Mean | 2.23 | 1 | 38.52 | 93 | 32.82 | 208 | 19.93 | 196 | 6.51 | 93 |
|  | SD | 1.93 | 1 | 9.81 | 46 | 7.62 | 90 | 5.41 | 100 | 4.68 | 89 |

|  |  | Forward displacement | | Backward displacement | | Left displacement | | Right displacement | |
| --- | --- | --- | --- | --- | --- | --- | --- | --- | --- |
|  |  | **% of time** | **Dist (m)** | **% of time** | **Dist (m)** | **% of time** | **Dist (m)** | **% of time** | **Dist (m)** |
| Defensive (N=374) | Mean | 36.23 | 383 | 16.24 | 140 | 22.10 | 176 | 25.44 | 232 |
|  | SD | 4.84 | 191 | 2.74 | 64 | 3.45 | 82 | 3.87 | 113 |
| Offensive (N=359) | Mean | 41.15 | 276 | 8.26 | 69 | 19.58 | 96 | 25.08 | 149 |
|  | SD | 8.05 | 164 | 4.99 | 46 | 5.35 | 61 | 5.22 | 98 |
| Defensive transition (N=393) | Mean | 46.44 | 188 | 16.79 | 53 | 15.09 | 48 | 21.67 | 84 |
|  | SD | 13.52 | 112 | 9.24 | 42 | 7.35 | 36 | 9.78 | 68 |
| Offensive  Transition (N=392) | Mean | 51.67 | 321 | 14.20 | 39 | 15.08 | 79 | 24.99 | 146 |
|  | SD | 12.59 | 170 | 4.46 | 38 | 8.04 | 73 | 11.34 | 102 |

N, number of players; SD, standard deviation; dist, distance covered.
