# Supplementary material for: A deep dive into the use of local positioning system in professional handball: Automatic detection of players’ orientation, position and game phases to analyse specific physical demands
Source: PLoS One. 2023 Aug 16;18(8):e0289752. doi: 10.1371/journal.pone.0289752 (PMC10431627; doi:10.1371/journal.pone.0289752)
Supplement: S5 Table — (DOCX) [file pone.0289752.s005.docx]

**S5 Table. Dunn's post-hoc test for the variable Distance normalised for game phases factor.**

|  | | | | | | | | | | | | | |
| --- | --- | --- | --- | --- | --- | --- | --- | --- | --- | --- | --- | --- | --- |
| **Comparison** | | **z** | | **W _i_** | | **W _j_** | | **p** | | **p _bonf_** | | **p _holm_** | |
| Def_Transition - Defensive play |  | 29.558 |  | 1178.950 |  | 206.361 |  | < .001 | *** | < .001 | *** | < .001 | *** |
| Def_Transition - Off_Transition |  | -2.084 |  | 1178.950 |  | 1246.765 |  | 0.019 | * | 0.112 |  | 0.019 | * |
| Def_Transition - Offensive play |  | 17.367 |  | 1178.950 |  | 601.902 |  | < .001 | *** | < .001 | *** | < .001 | *** |
| Defensive play - Off_Transition |  | -31.601 |  | 206.361 |  | 1246.765 |  | < .001 | *** | < .001 | *** | < .001 | *** |
| Defensive play - Offensive play |  | -11.772 |  | 206.361 |  | 601.902 |  | < .001 | *** | < .001 | *** | < .001 | *** |
| Off_Transition - Offensive play |  | 19.397 |  | 1246.765 |  | 601.902 |  | < .001 | *** | < .001 | *** | < .001 | *** |
|  | | | | | | | | | | | | | |
| * p < .05, *** p < .001 | | | | | | | | | | | | | |
