# Supplementary material for: A deep dive into the use of local positioning system in professional handball: Automatic detection of players’ orientation, position and game phases to analyse specific physical demands
Source: PLoS One. 2023 Aug 16;18(8):e0289752. doi: 10.1371/journal.pone.0289752 (PMC10431627; doi:10.1371/journal.pone.0289752)
Supplement: S6 Table — (DOCX) [file pone.0289752.s006.docx]

**S6 Table. Dunn's post-hoc test for the variable Accel’Rate normalised for game phases factor.**

|  | | | | | | | | | | | | | |
| --- | --- | --- | --- | --- | --- | --- | --- | --- | --- | --- | --- | --- | --- |
| **Comparison** | | **z** | | **W _i_** | | **W _j_** | | **p** | | **p _bonf_** | | **p _holm_** | |
| Def_Transition - Defensive play |  | 25.959 |  | 1031.043 |  | 209.023 |  | < .001 | *** | < .001 | *** | < .001 | *** |
| Def_Transition - Off_Transition |  | -4.734 |  | 1031.043 |  | 1179.190 |  | < .001 | *** | < .001 | *** | < .001 | *** |
| Def_Transition - Offensive play |  | 14.174 |  | 1031.043 |  | 577.448 |  | < .001 | *** | < .001 | *** | < .001 | *** |
| Defensive play - Off_Transition |  | -30.619 |  | 209.023 |  | 1179.190 |  | < .001 | *** | < .001 | *** | < .001 | *** |
| Defensive play - Offensive play |  | -11.375 |  | 209.023 |  | 577.448 |  | < .001 | *** | < .001 | *** | < .001 | *** |
| Off_Transition - Offensive play |  | 18.791 |  | 1179.190 |  | 577.448 |  | < .001 | *** | < .001 | *** | < .001 | *** |
|  | | | | | | | | | | | | | |
| *** p < .001 | | | | | | | | | | | | | |
